# Supplementary figures and images for: Effects of Thermoforming on the Mechanical, Optical, Chemical, and Morphological Properties of PET-G: In Vitro Study
Source: Polymers (Basel). 2024 Jan 10;16(2):203. doi: 10.3390/polym16020203 (PMC10820839; doi:10.3390/polym16020203)

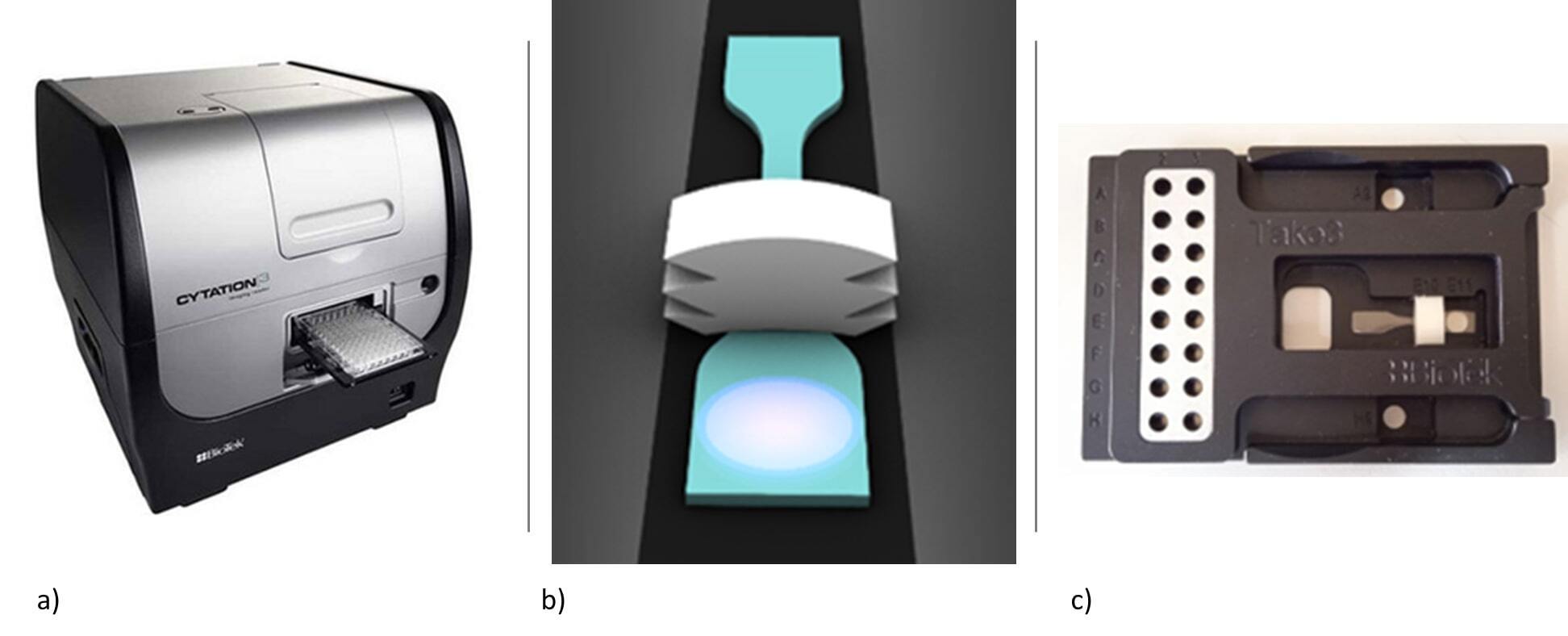

Supplement: Supplementary file 1 [file polymers-16-00203-s001.zip › S1(a-b-c).JPG.jpg]

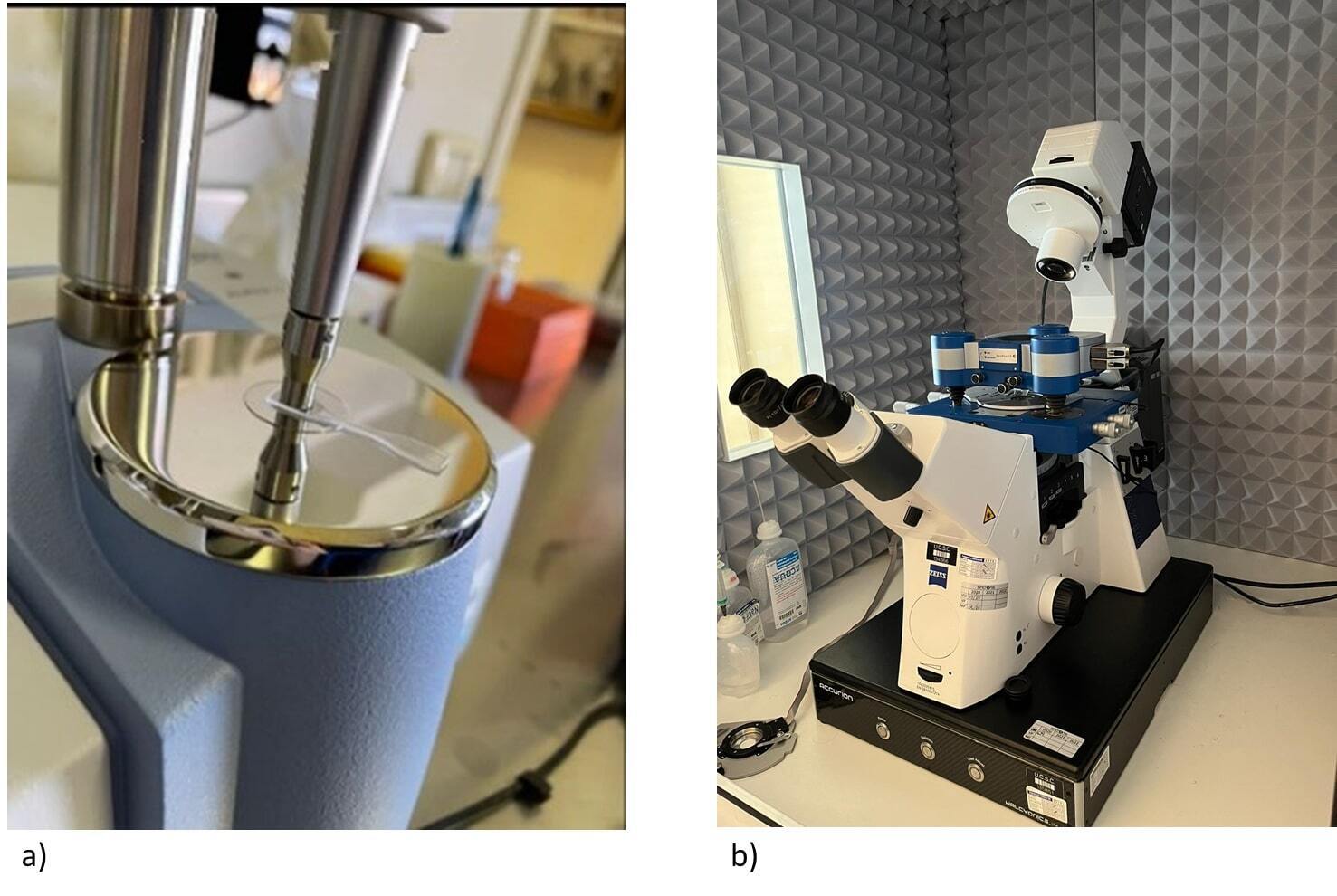

Supplement: Supplementary file 1 [file polymers-16-00203-s001.zip › S2(a-b).JPG.jpg]

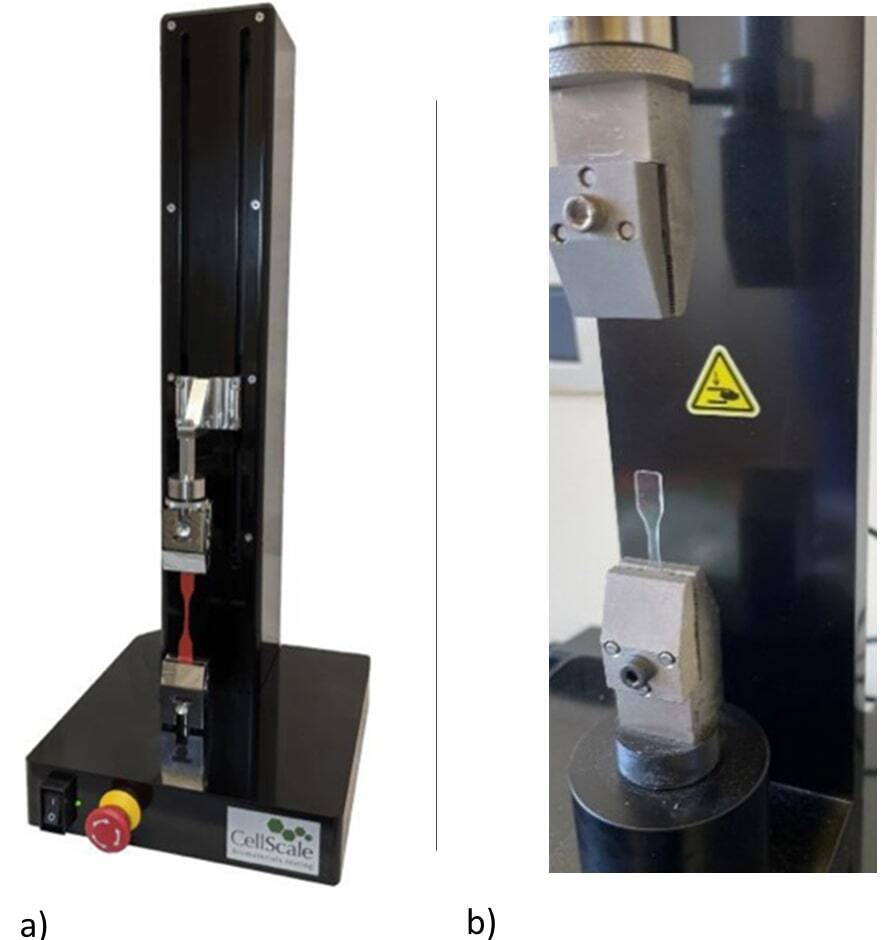

Supplement: Supplementary file 1 [file polymers-16-00203-s001.zip › S3(a-b).JPG.jpg]
